# Precision Nutrition in NAFLD: Effects of a High-Fiber Intervention on the Serum Metabolome of NAFD Patients—A Pilot Study

**DOI:** 10.3390/nu14245355

**Published:** 2022-12-16

**Authors:** Ewa Stachowska, Dominika Maciejewska-Markiewicz, Joanna Palma, Karolina Anna Mielko, Badr Qasem, Katarzyna Kozłowska-Petriczko, Marcin Ufnal, Katarzyna Ewa Sokolowska, Victoria Hawryłkowicz, Patrycja Załęska, Karolina Jakubczyk, Ewa Wunsch, Karina Ryterska, Karolina Skonieczna-Żydecka, Piotr Młynarz

**Affiliations:** 1 Department of Human Nutrition and Metabolomics, Faculty of Health Science, Pomeranian Medical University in Szczecin, 71-460 Szczecin, Poland; 2 Department of Biochemical Science, Faculty of Health Science, Pomeranian Medical University in Szczecin, 70-204 Szczecin, Poland; 3 Department Biochemistry, Molecular Biology and Biotechnology, Faculty of Chemistry, Wrocław University of Science and Technology, 50-370 Wrocław, Poland; 4 Department of Translational Medicine, Pomeranian Medical University in Szczecin, 70-204 Szczecin, Poland; 5 Laboratory of Centre for Preclinical Research, Department of Experimental Physiology and Pathophysiology, Faculty of Medicine and Dentistry, Medical University of Warsaw, 02-097 Warsaw, Poland; 6 Independent Clinical Epigenetics Laboratory, Pomeranian Medical University in Szczecin, Unii Lubelskiej 1, 71-252 Szczecin, Poland

**Keywords:** NAFLD, metabolome, SCFA, BCFA

## Abstract

Non-alcoholic fatty liver disease (NAFLD) is associated with dysfunction of the intestinal microbiota and its metabolites. We aimed to assess whether replacing bread with high-fiber buns beneficially changes the metabolome in NAFLD patients. This study involved 27 adult patients with NAFLD validated by FibroScan^®^ (CAP ≥ 234 dB/m). Patients were asked to replace their existing bread for two meals with high-fiber buns. In this way, the patients ate two rolls every day for 2 months. The following parameters were analysed (at the beginning and after 2 months): the anthropometric data (BIA), eating habits (24 h food recalls), gut barrier markers (lipopolysaccharide S and liposaccharide binding protein (LPS, LBP)), serum short-chain fatty acids (SCFAs) and branched chain fatty acids (BCFAs) by GC/MS chromatography, as well as serum metabolites (by ^1^H NMR spectroscopy). After 2 months of high-fiber roll consumption, the reduction of liver steatosis was observed (change Fibroscan CAP values from 309–277 dB/m). In serum propionate, acetate, isovaleric, and 2-methylbutyric decrease was observed. Proline, choline and one unknown molecule had higher relative concentration in serum at endpoint. A fiber-targeted dietary approach may be helpful in the treatment of patients with NAFLD, by changing the serum microbiota metabolome.

## 1. Introduction

Precise nutrition with the advent of multiple “-omics”, including metabolomics, might confer huge benefits for clinicians to evaluate the efficacy of certain dietary interventions [1]. One of the diet-related diseases that seems to be an excellent target for the principles of precision nutrition is non-alcoholic fatty liver disease (NAFLD) [2]. This disease is the most common chronic liver condition in Western countries [3]. The prevalence of NAFLD is estimated to be as much as 24.1%, with differences among gender, age and ethnicity [4]. NAFLD refers to a spectrum of liver disorders, closely associated with obesity and metabolic syndrome, characterized by abnormal fat accumulation in the liver, inflammation, and consequently, hepatocyte dysfunction [5]. The pathogenesis of NAFLD also includes severe dysfunction of the gut microbiome, referred to as intestinal dysbiosis [6]. Dysbiosis refers to an unfavorable change in the composition of bacteria in the gut (e.g., decreased α diversity) or/and changes in the amount of synthesized bacterial metabolites that are key to liver function (e.g., short-chain fatty acids (SCFAs), choline, or secondary bile acids) [7]. Dysbiosis exerts a negative impact on the functioning of the intestinal barrier, leading to the phenomenon known as leaky gut syndrome, which in turn causes harmful bacterial translocation into the portal vein (endotoxemia) and induces inflammation in the liver [8,9]. Overall, dysbiosis and increased intestinal permeability are key factors involved in NAFLD development and in the acceleration of its progression [7].

Diet exerts a significant impact on the composition and diversity of the intestinal microbiota (diversity, beneficial bacteria abundance), and thus may have a pivotal role for the course of endotoxemia in the NAFLD [10,11]. A Western-type diet (poor in fiber) destroys the balance within the intestinal habitat of microorganisms, and increases the synthesis of toxic metabolites (e.g., branched chain fatty acids (BCFA), pathogen-associated molecular patterns (PAMPs), lipopolysaccharides (LPS), ethanol) [7,12]. On the other hand, the administration of different types of fibers (complex, indigestible carbohydrates) boosts microorganism richness and the integrity of the gut barrier [8]. Fiber via saccharolytic fermentation is metabolized to SCFAs, which have many metabolically beneficial functions for both the bacteria and the host. Among others, SCFAs are an energy source for commensal gut bacteria and body weight regulators (through increased secretion of gut peptides GLP-1 and GLP-2) [7].

A meta-analysis published in 2019 demonstrated that fiber administration at doses of 10 to 16 g per day (study duration between 10 and 12 weeks) resulted in favorable changes in liver-related biomarkers (insulin resistance index (HOMA-IR), alanine aminotransferase (ALT), and aspartate aminotransferase (AST) levels) [13].

There are very few research results indicating whether administration of fiber has any beneficial effect on the serum bacterial metabolome.

Markers of unfavorable changes in the bacterial metabolome may include a decrease of blood concentrations of SCFA in favor of an increase in BCFA and LPS [14]. Similar biochemical patterns in patients with metabolic syndrome are exponents of excessive proteolytic fermentation in the intestine and serve as markers of disease severity [15,16].

To date, relatively few papers have been published on changes in the serum metabolome of patients with NAFLD [17]. There is a lack of work aimed at assessing the whole metabolome of gut bacteria in serum induced by dietary intervention in NAFLD. Therefore, the primary objective of this work is to answer the question on how a dietary intervention with a high-fiber rolls introduced into the diets of patients with NAFLD affect their gut bacteria metabolome studied in serum.

## 2. Materials and Methods

### 2.1. Study Design, Study Population Recruitment and Dietary Information

A NAFLDroll (ClinicalTrials.gov Identifier:NCT04520724) trial was conducted among individuals of the Sonomed Medical Centre in Szczecin (Poland). All patients were recruited for the study by a hepatologist between July 2019 and November 2019. Everyone in the study group was traditionally carnivorous. Comorbidities were Hashimoto’s disease (*n* = 1) and hypertension (*n* = 4). Medications taken were Euthyrox and Letrox, and for hypertension, Concor and Bisocard.

At baseline, patients were acquainted with the principles of proper nutrition (the principles of the Mediterranean diet) by licensed dietitians. After qualification, patients gained access (3 times per week) to the special high-fiber rolls (buns). The company producing high-fiber rolls (EKOTAST, Poland) supplied them to selected shops. Patients were asked to replace their bread for two meals with the aforementioned buns. In this way, the patients ate 2 rolls every day for 2 months. Adherence to the intervention was verified by checking collection of bread rolls through a telephone call to the employees of the shops involved in the project.

**Figure 1:**
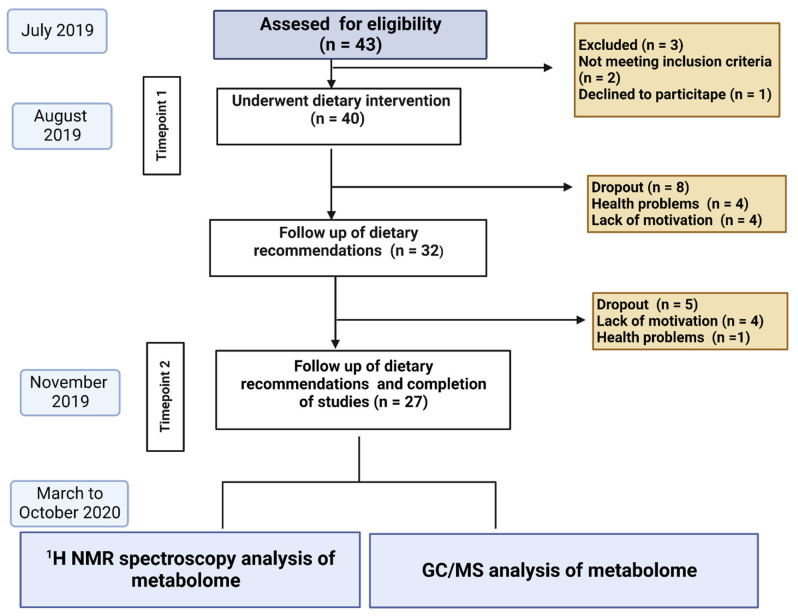
Description of the tests performed. GC/MS chromatography (GC/MS), Nuclear magnetic resonance spectroscopy ^1^H NMR.

All measurements and sample collection was performed two times during the study: at baseline (Timepoint 1) and after 60 days (Timepoint 2) (Figure 1).

In this study, we recommended moderate activity and we advised our patients not to change their physical activity during the study.

### 2.2. Intervention

A group of 43 eligible adult participants (13 woman, 14 men) was initially enrolled in the study on the basis of previously established clinical diagnosis of NAFLD. In all patients, careful clinical evaluation was performed by an experienced hepatologist, including a physical examination, abdomen ultrasound, and Fibroscan examination. In two patients, a controlled Attenuation Parameter (CAP) measurement by Fibroscan did not confirm liver steatosis (CAP cut-off 234 dB/m), thus these individuals were excluded from the study.

In the next step, the patients were evaluated for the exclusion criteria: infection with either HBV (Hepatitis B Virus) or HCV (Hepatitis C Virus); extremal obesity (body mass index (BMI) > 40 kg/m^2^); high levels of physical activity (>3000 kcal/week in leisure-time physical activity); planned changes in physical activity during the study period; and not being able to attend control visits. Additionally, we did not include patients practicing veganism, vegetarianism, or with a need for other special diets; the excessive consumption of alcohol (>30 g in men, and 20 g in women per day); drug addiction, and any condition that could limit the mobility of the participant.

Comorbidities in the group of patients that did not exclude them from dietary interventions were hypertension, hypothyroidism, Hashimoto’s disease, and degenerative spine disease.

None of the patients met the exclusion criteria; however, one patient declined to participate in the dietary intervention, therefore 40 participants were finally included in the study.

In the first period, 8 patients dropped out from the study (4 because of health problems not associated with the intervention, 4 because of the lack of motivation), and another 5 patients dropped out in the second study period (4 because of the lack of motivation, 1 because of health problems not associated with the intervention). Taken together, the drop-out rate was 13/40 (32.5%). As per the authors’ opinion, lack of motivation might have been associated with waiting for the implementation of pharmacological treatment, which gives faster results than dietotherapy. Twenty-seven participants completed the study and were finally analysed.

The study protocol was approved by the ethics committee of the Pomeranian Medical University (Szczecin, Poland, KB-0012/131/19) and conformed to the ethical guidelines of the 1975 Declaration of Helsinki. The volunteers provided written informed consent before the study.

### 2.3. The Anthropometric Data

Anthropometric assessments were performed routinely during each of the timepoints. The study included measurements of height (m), body weight (kg), body composition expressed as: fat mass (percentage body fat, PBF) in %, lean mass in % and total body water (TBW) in %. All of the anthropometric parameters were measured with a multifrequency bioimpedance device, Tanita BC 601 (Tokyo, Japan).

### 2.4. Liver Stiffness and Steatosis Measurements

Liver stiffness (Vibration-Controlled Transient Elastography, or VCTE) and steatosis evaluation (Controlled Attenuation Parameter, or CAP) measurements were performed simultaneously by a single observer (hepatologist) using FibroScan^®^ (Echosens, Paris, France) after at least 6 h of patients’ fasting at both timepoints. Measurements were made using both M and XL probes, depending on skin-to-liver capsule distance in the right lobe of the liver, through intercostal spaces, with the patient lying in a dorsal position with the right arm in maximal abduction. The final results of CAP and VCTE were the median value of 10 measurements expressed in dB/m and kPa, respectively. Only results with an interquartile range of ≤40% were considered reliable. The steatosis grades were established using the following cut-off values for low-, intermediate-, and high-grade steatosis (S1, S2, S3): 234, 269, and 301 dB/m, respectively [18].

### 2.5. Dietary Guidelines and Control Visits

Licensed nutritionists at the first timepoint gave an individual 30 min presentation on the principles of the Mediterranean diet, discussing its composition and benefits in detail. Each patient was given information to replace a customary bread (to be eaten twice a day—at breakfast and second breakfast) with special high-fiber rye rolls.

The composition of the rolls was as follows: rye flour type 2000 BIO, vital fiber (20% plantain, 80% psyllium) BIO, apple fiber BIO, ground milk thistle BIO, natural leaven from the fermentation of rye flour type 2000, and yeast. The fiber content in the rolls was determined according to the method described by AOAC [18]. Total insoluble and soluble dietary fiber was determined according to the enzymatic–gravimetric method using a Fibertec 1023 device (Tecator Tech., Höganäs, Sweden). The average fiber content in rolls was 6.6 ± 0.11 g/roll, fat 2.38 ± 0.11 g/roll, protein 20.4 ± 0.47 g/roll, and water 63.7 ± 0.77 g/roll.

A dietician estimated the consumption of fiber sources: pulse foods, cereals or grains, baked goods or pastries, nuts, potatoes, pasta and rice, fruits, and vegetables, as well as the total number of daily servings of vegetables, fruit, bread, dairy products, spreadable fats, eggs, snacks, as well as fats.

Dietary analysis of the daily intake of total fiber from the dietary records was achieved using nutrient analysis software (Dieta 6 software with the built-in database of the Polish National Institute of Nutrition, Poland).

### 2.6. Blood Sample Collection

The total blood samples were collected from each participant at each of the timepoints. The blood samples were taken after a minimum of 12 h of fasting at the laboratory of the Clinical Hospital No. 1 in Szczecin. In the blood measurements, the triglycerides, total cholesterol, LDL, HDL, glycemic parameters (glucose, insulin), and liver enzyme activity (GGTP, ALT, AST) were performed. Endotoxemia parameters, including LPS and LPB, were measured by ELISA according to the manufacturer’s protocol (EIA Lab, Wuhan, China) and LPB (Fine Test, Wuhan, China).

**Figure 2:**
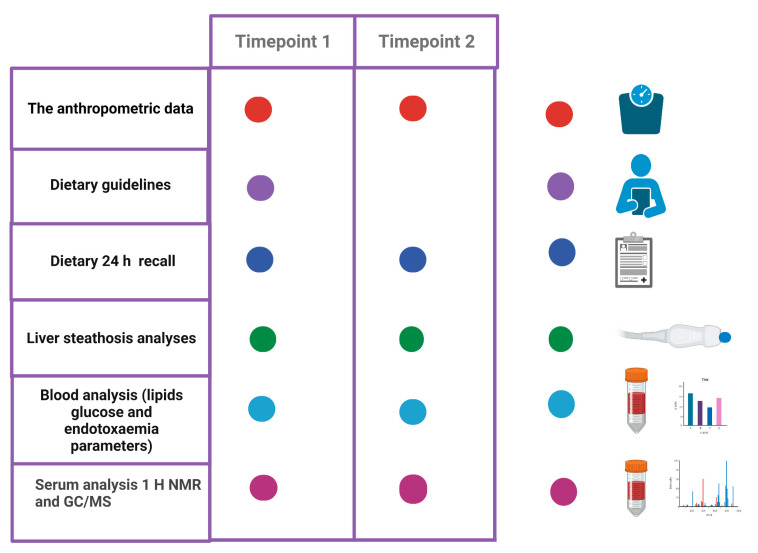
A scheme presenting outcome measures at study timepoints.

Blood samples for the bacterial metabolome study were collected into EDTA tubes and after centrifugation (1500× *g* × 10 min), and serum was stored at −80 °C until analysis. A summary of the activities is presented in Figure 2.

### 2.7. Outcome Measures

#### 2.7.1. Serum Short-Chain Fatty Acids (SCFAs) and Branched Chain Fatty Acids (BCFAs) in Plasma Analysis by Means of GC/MS Chromatography

Sample preparation was carried out in accordance with the ref. [19]. First, solutions of 400 mM 3 NPH and 240 mM EDC-6% pyridine were freshly prepared in 50% aqueous acetonitrile. The derivatization procedure was as follows: 40 µL of plasma and calibration samples were mixed with 80 µL methanol (containing internal standards) on a 96-well 2 mL sample plate. Afterwards, 20 µL of 3NPH solution and 20 µL of EDC-pyridine solution were added. The mixture was incubated in room temperature for 30 min. Next, the solution was diluted to 1 mL with 15% aqueous acetonitrile, centrifuged, and the aliquot was injected into an apparatus.

The instrumentation consisted of a Waters Acquity Ultra Performance Liquid Chromatograph coupled with a Waters TQ-S triple-quadrupole mass spectrometer. For the instrument control and data acquisition, Waters MassLynx software was used. Waters TargetLynx was used to process data.

The analytes separation was performed using a Waters BEH C18 column (1.7 µm, 2.1 mm × 50 mm) and Waters BEH C18 guard column (1.7 µm, 2.1 mm × 5 mm). Mobile phase A consisted of 1 mL of formic acid in 1 L water, and mobile phase B consisted of 1 mL of formic acid in acetonitrile. The flow rate of the mobile phase was set at 0.6 mL/min. The column temperature was 60 °C, and the autosampler was kept at 5 °C. The injection volume was 10 µL.

The mass spectrometer operated in multiple-reaction monitoring (MRM)—negative electrospray ionization (ESI) mode. For all analyzed compounds, the mass spectrometer optimized settings were as follows: capillary voltage = 2.25 kV, desolvation temperature = 550 °C, desolvation gas flow = 550 L/h, cone gas flow = 150 L/h, nebuliser gas pressure = 7.0 bar, source temperature = 150 °C. The MRM transitions, cone voltages, collision energies and retention times used in the described methods are presented in Table 1. The first MRM transition of each compound served as a quantitative transition, and the second as a confirmation transition.

The concentration of short-chain fatty acids was calculated using a calibration standard mix derived from a series of calibrator samples by spiking standard stock solutions into water. Calibration curves for SCFAs were generated by comparing a ratio of the peak area of the analyzed compound to the peak of the internal standard against known analyte concentrations. Mean R2 coefficients of calibration curves from six calibrators was not lower than 0.99. The method showed a good intra- and interassay precision below 10%.

##### H NMR Spectroscopy Analysis of the Bacterial Metabolites

Samples were thawed in an ice bath; 300 µL of serum was transferred to a new 1.5 mL tube and 600 µL of methanol was added to each sample (extraction 1:2). In the next step, vortexed samples were placed at −20 °C for 20 min. After that time, samples were centrifuged (1100 rpm, 30 min, 4 °C), and 700 µL of a clarified upper phase was transferred into a new tube. The extracts were evaporated in a vacuum centrifuge (Christ RVC 2–25 CDplus) (40 °C, 1500 rpm, 8 h). In the next step, 600 µL of PBS buffer (0.5 M, 20% D_2_O, pH = 7.0, 0.03 mM TSP) was added to each sample and mixed for 3 min. The obtained samples were centrifuged (1100 rpm, 10 min, 4 °C) and 550 µL was transferred into 5 mm NMR-tubes (5SP, Armar Chemicals) for ^1^H NMR measurements.

Standard one-dimensional ^1^H NMR experiments were performed on a Bruker AVANCE II 600.58 MHz spectrometer equipped with a 5 mm TBO probe at 300 K. All 1D ^1^H NMR spectra were carried out using the *cpmgpr1d* (in Bruker notation) pulse sequence with suppression of water resonance by presaturation. Acquisition parameters were as follows: spectral width, 20.01 ppm; the number of scans, 128; acquisition time, 2.72 s per scan; relaxation delay, 3.5 s; O1P = 4.721 ppm was set for residual water signal presaturation; 0.001 s spin echo delay and 64 K time-domain points. Before Fourier transformation, the FIDs were multiplied by an exponential function equivalent to that of a 0.3 Hz line-broadening factor. The spectra were referenced to the TSP resonance at 0.0 ppm and manually corrected for phase and baseline (MestReNova v. 11.0.3). For ^1^H NMR signal identification, the Chenomx NMR Analysis Software (v. 8.5, Chenomx Inc.) was used. For statistical analysis, only one signal for each metabolite was used.

### 2.8. Data Processing and Multivariate Statistical Data Analysis

For GC/MS, all spectra were exported to Matlab (Matlab v. 8.3.0.532) for preprocessing. Regions affected by solvent suppression were excluded (4.68–5.05 ppm). Signals alignment was performed by the correlation of optimized warping (COW) and interval correlation shifting (icoshift) algorithms. The spectra consisted of 8.910 data points and were normalized using the probabilistic quotient method (PQN) to overcome the issue of dilution, where all spectra were used as a reference group.

The multivariate and statistical data analysis was performed on a set of the 30 (+3 unknown) assigned metabolites. The relative concentration of metabolites measured by NMR was obtained as the sum of data points in the data matrix for the non-overlapping resonances (or a part of the partly overlapping resonances) range. The input for SIMCA software (v 15.02, Sartorius Stedim Data Analytics AB) was a transformed data matrix. The data sets were scaled using UV scaling before the chemometric analysis. For all samples, principal component analysis (PCA) and orthogonal partial least-square discriminant analysis (OPLS-DA) was carried out. The OPLS-DA model reliability was tested with CV-ANOVA at the level of significance of α < 0.05. Univariate analysis was performed in a R 4.1.3. environment with use of Student’s *t*-test (equal/unequal variance) for data originating from a normal distribution and using the Mann–Whitney–Wilcoxon test for data that did not meet these requirements. Normality of distribution was assessed by the Shapiro–Wilk test. The correction for multiple comparisons was preceded with the Benjamini–Hochberg procedure (FDR). All univariate statistics were carried out at the level of significance of α ≤ 0.05. Post hoc power analysis was performed using G*Power software (Dusseldorf, Germany) [20]. 

## 3. Results

### 3.1. The Dietary Intervention Contributed to a Reduction in Lipid Deposits in the Liver and a Reduction in Body Weight

Reconstructed from the diaries, the caloric content of the diet and the fiber content in the two timepoints did not differ significantly (Table 2). After 2 months of roll consumption, a trend in reduction in body mass index (BMI) was achieved among patients (29.5 kg/m^2^ vs. 28.9 kg/m^2^, adj. *p*-value = 0.057). However, this small reduction in BMI did not have a clear effect on other body composition parameters, such as body fat or lean body mass. Importantly, after 2 months of intervention, lipid deposits decreased significantly (adj. *p*-value = 0.04), which is reflected in the reduction of Fibroscan CAP values from 309 dB/m (range: 242–400) at T1 to 277 dB/m (range: 224–371). We here confirm that the decrease of liver steatosis grades was noted in 9 patients (S1 à S0: *n* = 2, S2 à S1: *n* = 3, S3 à S1: *n* = 3, S3 à S1: *n* = 1), and in 16 patients no change was noted regarding this parameter. However, a high grade of steatosis was still observed [21].

We considered the Fibroscan CAP value as primary outcome, and for this data we calculated the statistical power (baseline-endpoint). Using a Wilcoxon test for one sample case, the effect size was calculated to be 0.404. Taking into account a given sample size, α = 0.05, the β value was 0.79.

### 3.2. The Caloric and Fiber Content of the Diet Explain the Change in Serum Metabolites

In principle, our intervention was not to introduce caloric restrictions, but to introduce more fiber into the current diet, which was achieved by including high-fiber rolls in the diet. The caloric content of the diet, analyzed via 24 h recall diaries, is presented in Supplementary Table S1. The 24 h menus did not differ significantly in caloric content between the measurement points and with the mean indicating a hypocaloric nature of the diet (1464 kcal (775.5–2323.8)–T1 vs. 1364 (847.5–2051)–T2; adj. *p*-value = 0.18). It seems that the obtained result of the underestimated declaration of the calorific value of meals was underestimated by patients, which is a phenomenon present in the overweight or the obese [22]. On the other hand, the inclusion of high-fiber bread in the diet resulted in a 1.5-fold increase in the fiber content in the patients’ diet. From the initial amount of less than 18 g per day, the fiber content increased to 24 g (adj. *p*-value = 5.87 × 10^−5^ at the endpoint. This value still does not reach the value recommended for adult men aged 51–70 years old (30 g/day) but exceeds the requirement for a woman in this age range (~22 g/day) [23].

The increased satiety experienced during an increase in dietary fiber may explain this modest weight loss noted at the T2 assessment [23]. We showed that during the 2-month follow-up period, our patients reduced their BMI by 1 kg/m^2^ (Table 1). There is a clear trend towards weight reduction that might be sufficient to improve the parameters of fatty liver by 32 dB/m, as measured by the Fibroscan CAP method. At the same time, no negative impact of consuming an increased amount of fiber on the release parameters, as well as lipid and glycemic parameters in the group of patients, was observed (Table 1).

### 3.3. The Use of Precise Nutrition Had an Effect on the Concentration of SCFA and BCFA in the Serum

The obtained results seem to confirm the change in the characteristics of fatty acids synthesized in the intestines by the intestinal microbiome. We demonstrate a decrease in the content of two major SCFAs, propionate and acetate (but not butyrate) in the serum (Table 2).

Apart from these major SCFAs, the intestinal microbiota also produced considerably lower amounts of isobutyric, isovaleric, and 2-methylbutyric acids, commonly known as branched short-chain fatty acids (BCFA) [24].

The largest changes in the content were noted for BCFA—methylbutyric, valeric, caproic, isovaleric acids and isocaproic acid/4-methylvaleric acid, which indicates a potential reduction in proteolytic fermentation in the intestines introduced via dietary intervention.

Importantly, the intervention had a positive effect on gut-derived endotoxemia—although among the parameters we measured (LPB and LPS), only the LPB was significantly reduced (Table 1). Consequently, we assumed that a decrease in LPB content and a concomitant lack of increase in serum LPS content indicates a slight improvement in liver function [25,26].

### 3.4. Metabolites Identification ^1^H NMR Spectrum

In total, 30 metabolites were identified (acetate, acetone, alanine, choline, creatinine, ethanol, formate, glucose, glutamine, glycerol, glycine, homoserine, imidazole, isobutyrate, isoleucine, lactate, leucine, lysine, methanol, methionine, phenylalanine, proline, pyroglutamate, pyruvate, succinate, tryptophan, tyrosine, uracil, valine, π-methylhistidine; Table 3). Additionally, three signals were assigned on the ^1^H NMR spectrum as unknown (which cannot be attributed to metabolites based on data appearing in spectroscopic databases). However, these metabolites have significant signal intensities and were also used in all analyses. All assignments were verified using the human metabolome database (HMDB).

The results of statistical analyses are presented below in Table 3.

Among statistically important metabolites, only two had lower relative concentration in samples from Timepoint 1 (proline, choline) and one metabolite had higher relative concentration in samples from Timepoint 2.

## 4. Discussion

An interesting direction in the use of metabolomics is the search for molecules useful in the early diagnosis of metabolic diseases of the liver. Pioneering research in this area indicates changes in the metabolome during the development of non-alcoholic steatosis or the appearance of an inflammatory process in the liver (NASH) [27]. Researchers have been able to show that in patients suffering from steatosis and inflammation, the metabolism of amino acids, fatty acids and vitamins is significantly changed [28].

In this study, we wanted to check whether a simple dietary intervention consisting of a radical increase in dietary fiber will bring about measurable changes in the metabolome that may indicate an improvement in liver function. It seems that the aim of the study has been achieved and we have obtained a significant reduction in the content in the circulation of these metabolites that may be responsible for the progression of steatosis.

The content of selected amino acids in serum has been linked to the pathogenesis of NAFLD and the progression of the disease to hepatitis (NASH) and liver fibrosis [29,30]. It has been shown that the content of most amino acids increases in parallel with the development of insulin resistance and the aggravation of the metabolic syndrome in obese people with NAFLD [31], while the process of liver fibrosis is associated with changes in the concentration of glutamate and glycine (the content of which in the blood did not change significantly in our study). We also did not observe changes in the concentration of BCAA (isoleucine, leucine, and valine) in the serum, which are recognized predictors of fatty liver disease [28]. The reason seems quite obvious—the dietary intervention we introduced (increasing the content of fiber in the diet) was too “subtle” metabolically—it was aimed at rebuilding the bacterial metabolome. However, the obtained results indicate that without the parallel introduction of caloric restrictions that would force the reduction of patients’ body weight, the observation of changes in the concentration of amino acids crucial for the progression of NAFLD (e.g., BCAA) is not possible. It seems that the study model adopted by us was not radical enough or took too little time to obtain a metabolic effect. However, the study model was sufficient to observe changes in the metabolome of the gut microbiome. Bacterial metabolome presumably indicates a reduction in the content of both SCFA and BCFA, which may indicate a better use of SCFA (especially propionate as fuel). On the other hand, a significant decrease in the content of most BCFA may be the result of a reduction in the strength of bacterial proteolytic fermentation in patients.

The introduction of a daily additional portion of fiber to the patients’ diet led to a change in the concentration of three metabolites, two of which—choline and proline—significantly reduced the concentration in the blood, and the concentration of a third unknown increase in the serum. Choline is a metabolite whose reduction was noticeable in all patients. Choline is a key ingredient for liver metabolism. In the liver, choline is used for the production of phospholipids, as a substrate for oxidation and as a donor of methyl groups [32]. A particularly important metabolite of choline in the liver is phosphatidylcholine, used for the synthesis of very low-density lipoprotein (VLDL) and their export [33] and for the metabolism of bile salts [34,35]. Dietary choline deficiencies have been linked to pathogenesis of fatty liver in rodents [36,37]. On the other hand, there are doubts on the intensity of such process in humans. It is also difficult for us to unequivocally determine whether the reduction of choline in the serum of patients observed by us is a disturbing symptom and indicative of a progressive choline deficiency in the diet or a result of another phenomenon. Choline is found in a variety of foods, but it is particularly abundant in egg yolks and animal sources of protein. The diet recommended by nutritionists at the beginning of the study recommended limiting animal sources of fat and protein [37]. The result obtained may therefore be an emanation of wider changes in the way patients eat—including the reduction of animal products by our patients.

Proline is one of those amino acids of which an increase in the serum may indicate the progression of the fatty liver process [29]. In a study by a Japanese team, 797 participants were examined using transient elastography, whereby 251 of them were diagnosed with NAFLD, 33 with fibrosis. Among the subjects, significant differences in the serum levels of most amino acids were noted—including proline being an indicator of the progression of fatty liver and fibrosis. Serum PRO concentration increased from 129 (nmol/mL) in the healthy liver group to 141 (nmol/mL) in NAFLD patients (*p* < 0.001) and from 129 to 152 (nmol/mL) in fibrosis patients (*p* < 0.001) (0.002) [29]. The increase in blood proline concentration was associated with a slight increase in insulin resistance expressed by an increase in the HOMA-IR index. In our experiment, the proline level significantly decreased, directly mirroring the healing fibre effect on liver condition.

The key element influencing the condition of the liver may be the intestinal microbiota. Some researchers demonstrated data on liver function in regard to bacterial metabolites and liver function [21]; however there is still no consensus among clinicians [38]. Such associations have been found for small intestinal bacterial overgrowth (SIBO) in which endotoxemia and influx of pathogenic bacterial metabolites may explain the progression of fatty liver [39]. Moreover, microbiota metabolites, such as SCFA, choline metabolites, or secondary bile acids regulate metabolic pathways in the liver. Damage of the intestinal barrier can cause bacterial translocation into the blood and the portal vein, and induce inflammation in the liver [8]. Low dietary fiber intake is associated with higher rates of microbiota-associated chronic diseases, such as obesity. Low-fiber diets alter not only the microbial composition, but also the availability of metabolic end-products derived from fermentation of fiber.

SCFAs are major gut microbiota metabolites originating from a dietary source [40]. The main products of saccharolytic fermentation are acetic, propionic and butyric acid. SCFAs are natural ligands for G receptors (GPCR -41 and GPCR-43) found in many types of cells and tissues (e.g., enteroendocrine and immune cells) [41]. SCFAs are responsible for maintaining the integrity of the intestinal barrier, nourishing the intestinal cells, and activating the goblet cells to produce the right amount of mucus. Short-chain fatty acids appear to play an important role in regulating the integrity of the epithelial (intestinal) barrier through coordinated regulation of tight junction proteins (TJPs). The increased permeability of this barrier is related to the translocation of the bacteria and/or their cell wall components (e.g., lipopolysaccharide, LPS) into the circulation. This situation, commonly known as “leaky gut syndrome”, triggers an inflammatory cascade—first within the intestinal barrier, and then systemically. Such low-level inflammation stimulated by an inappropriate lifestyle, stress, or lack of physical activity is associated with obesity and insulin resistance [42]. Decreased levels of propionate production in NAFLD may result in increased intestinal permeability, with an increased risk of bacterial and LPS translocation into the systemic circulation [43] of the gut, where propionate as well as butyratre (that concentration did not change in this study) may reduce local inflammation, prevent progression of the inflammatory response into the systemic circulation, and may also promote tight junction function and gut integrity [44]. SCFAs are partially metabolized in the intestine, enter the gluconeogenesis pathway, and nourish the intestinal cells. However, some of these acids are absorbed and enter the liver [45]. After dietary intervention, we noticed declines in the major SCFAs, that is, propionic acid. With a properly functioning gut–liver axis, these acids should be used in the intestine and liver tissue in the main measure, and their presence in the blood should be temporary. Therefore, a reduction in the concentration of important SCFAs may indicate an improvement in the function of the gut–hepatic axis, although this hypothesis requires confirmation.

## 5. Conclusions

Clinicians and health science researchers working with patients emphasize the difficulty in adherence to recommended diets and physical activity. It has been shown that maintaining a healthy lifestyle followed by a well-balanced diet is unattainable for most patients, and only 50% of patients tend to maintain a 7% weight reduction after 12 months. However, weight reduction is our only way to achieve the likely changes in the metabolome. Every simple action—like recommending a certain type of food (like high-fiber rolls) is an interesting and valuable perspective. The obtained data seem to indicate that the fiber-targeted dietary approach may be helpful in the treatment of patients with NAFLD. The introduction of increased fiber in the diet without caloric reduction did not have a strong effect on the serum metabolome. It seems that a hypocaloric diet or a fiber intervention applied for a longer period of time than 2 months is required to obtain a clearer effect. However, the results need to be replicated in a larger population. Further study is warranted. 

## Figures and Tables

**Table 1:** Data on measurements taken in the study at baseline and endpoint.

Parameter (*n* = 27)	T1 Median (Range)	T2 Median (Range)	FDR Corrected *p*-Value
Anthropometry			
Age	51.1 (29–68)	-	
BMI (kg/m^2^)	29.5 (23.2–35.7)	28.9 (22.8–35.2)	0.057
Body weight (kg)	87.3 (60.3–115.6)	85.2 (59–113.9)	0.35
Visceral obesity (kg)	10 (5–18)	10 (4–18)	0.79
PBF (%)	29.8 (15.2–43.8)	27.4 (11.7–43.6)	0.18
Lean mass (kg)	55.9 (39.5–76.7)	56.8 (38.4–76)	1
TBW (%)	50.9 (41.9–60.2)	51.8 (42–63.4)	0.24
Dietary parameters			
Energy (kcal/day)	1464 (775.5–2323.8)	1364 (847.5–2051)	0.18
Total fiber in diet with rolls (g/day)	18.7 (10–33.6)	27.4 (17.3–44.8)	5.87 × 10^−5^
Liver function and metabolites			
Fibroscan CAP (dB/m)	309.3 (242–400)/95% CI: 290.40–328.14	277 (224–371)/95% CI: 274.94–310.68	0.04
Fibroscan (VCTE)	6 (3.9–9.4)/95% CI: 5.43–6.67	5.3 (3.6–9.7)/95% CI: 5.02–6.13	0.20
GGTP (IU/L) Norm (40–60 IU/L)	29 (12–100)	30 (11–102)	0.18
ALT (IU/L) Norm 35–40 IU/L	34 (11–136)	35 (11–86)	1
AST (IU/L) Norm (5 do 40 IU/L)	26 (11–52)	24 (13–40)	1
Endotoxemia parameters			
LPS (pg/mL)	96 (13–8856)	153 (0–481)	1
LBP (pg/mL)	25 (19–31)	23 (20–28)	0.004
Lipids and glucose metabolism			
Glucose (mg/dL) Norm (70–99 mg/dL)	93.3 (80.4–276.5)	96.1 (76.3–272.6)	0.63
Insulin (mIU/L) Norm (<25 mIU/L)	21.1 (6.7–152)	18.5 (4.3–129)	0.52
HDL mg/dL Norm (>50 mg/dL)	45.2 (25–71.3)	44.9 (25.8–77.5)	0.36
LDL (mg/dL) Norm (<115 mg/dL)	132.3 (43.5–282.2)	114.4 (47.9–258.3)	0.06
Total cholesterol (mg/dL) Norm (<190 mg/dL)	195.2 (110–394.4)	178.2 (98–340.2)	0.04
Triglycerides (mg/dL) (Norm < 150 mg/dL)	191.5 (76.1–700.5)	150.5 (50.9–452)	0.14

PBF—percentage of body fat, TBW—total body water, GGTP gamma-glutamyl transferase, ALT—Alanine transaminase, AST—Aspartate transaminase, LPS—Lipopolysaccharides, LBP—Lipopolysaccharide (LPS)—binding protein, CI—confidence interval.

**Table 2:** The content of fatty acids in serum. Data obtained by the GC-MS method.

Parameter (*n* = 27)	Timepoint 1	Timepoint 2	FDR Corrected *p*-Value
2_MeB	0.13 (0–0.67)	0 (0–0.29)	0.0013
VA	0.06 (0–0.29)	0 (0–0.05)	0.0056
CA	0.55 (0.33–1.29)	0.58 (0.34–0.88)	0.52
ICA	0 (0–0.26)	0 (0–0.15)	0.026
IVA	0.38 (0–1.53)	0.23 (0–0.9)	0.056
BA	0.44 (0.14–1.65)	0.31 (0.07–1.21)	0.033
PA	1.15 (0.41–2.59)	0.81 (0.5–2.38)	0.036
IBA	0.08 (0–0.49)	0.1 (0.05–0.26)	0.52
AA	26.13 (8.66–47.25)	21.95 (6.43–52.14)	0.56
MeVA	0 (0–0.13)	0 (0–0)	NA

AA—acetic acid (C2); PA—propionic acid (C3); IBA—isobutyric acid (C4); BA—butyric acid (C4); 2MeB—2-methylbutyric acid (C5); IVA—isovaleric acid (C5); VA—valeric acid (C5); ICA—isocaproic acid/4-methylvaleric acid (C6); and CA—caproic acid (C6).

**Table 3:** Summary statistical analysis—comparison at Timepoints 1 and 2.

Metabolite	Mean Relative Concentration T1	Mean Relative Concentration T2	RSD T1 [%]	RSD T2 [%]	*p*-Value	FDR Corrected *p*-Value
choline	0.58	0.48	27.13	19.90	5.41 × 10^−3^	7.14 × 10^−2^
proline	0.29	0.26	18.44	24.11	2.17 × 10^−2^	1.79 × 10^−1^
Unk_2	0.69	0.80	24.04	11.56	6.49 × 10^−3^	7.14 × 10^−2^
acetate	0.36	0.34	31.15	31.08	6.00 × 10^−1^	7.34 × 10^−1^
acetone	0.06	0.05	54.55	46.04	3.85 × 10^−1^	7.34 × 10^−1^
alanine	1.96	2.02	18.74	18.20	5.55 × 10^−1^	7.34 × 10^−1^
creatinine	0.28	0.30	24.95	22.47	2.39 × 10^−1^	6.06 × 10^−1^
ethanol	0.39	0.43	36.82	51.20	6.23 × 10^−1^	7.34 × 10^−1^
formate	0.06	0.05	29.16	30.71	5.93 × 10^−2^	3.69 × 10^−1^
glucose	3.21	3.16	25.13	21.16	8.44 × 10^−1^	8.44 × 10^−1^
glutamine	0.56	0.60	22.31	20.69	2.13 × 10^−1^	6.06 × 10^−1^
glycerol	0.10	0.12	37.99	31.79	1.01 × 10^−1^	3.69 × 10^−1^
glycine	0.89	1.01	28.71	25.34	9.12 × 10^−2^	3.69 × 10^−1^
homoserine	0.28	0.27	18.84	23.27	3.25 × 10^−1^	7.15 × 10^−1^
imidazole	0.27	0.26	15.80	15.59	4.92 × 10^−1^	7.34 × 10^−1^
isobutyrate	1.37	1.34	13.29	18.12	5.44 × 10^−1^	7.34 × 10^−1^
isoleucine	0.19	0.18	20.84	29.35	1.74 × 10^−1^	5.73 × 10^−1^
lactate	10.43	11.43	63.66	57.07	4.03 × 10^−1^	7.34 × 10^−1^
leucine	1.25	1.17	18.15	26.46	6.87 × 10^−2^	3.69 × 10^−1^
lysine	0.83	0.80	15.61	19.63	4.96 × 10^−1^	7.34 × 10^−1^
methionine	0.29	0.30	19.64	16.73	3.19 × 10^−1^	7.15 × 10^−1^
phenylalanine	0.31	0.30	10.45	10.69	2.37 × 10^−1^	6.06 × 10^−1^
pyroglutamate	0.22	0.24	22.65	19.63	9.92 × 10^−2^	3.69 × 10^−1^
pyruvate	0.26	0.24	49.97	51.61	5.60 × 10^−1^	7.34 × 10^−1^
succinate	0.23	0.21	55.43	62.05	4.81 × 10^−1^	7.34 × 10^−1^
tryptophan	0.20	0.20	32.25	27.07	6.02 × 10^−1^	7.34 × 10^−1^
tyrosine	0.49	0.50	14.21	15.71	7.07 × 10^−1^	7.78 × 10^−1^
Unk_1	0.18	0.17	44.74	48.79	7.31 × 10^−1^	7.78 × 10^−1^
Unk_3	0.10	0.10	33.85	29.53	7.71 × 10^−1^	7.95 × 10^−1^
uracil	0.17	0.17	16.81	23.13	6.52 × 10^−1^	7.42 × 10^−1^
valine	1.36	1.33	13.85	18.61	6.16 × 10^−1^	7.34 × 10^−1^
π-methylhistidine	0.12	0.11	36.11	48.86	5.23 × 10^−1^	7.34 × 10^−1^

RDS—relative standard deviation.

## Data Availability

Raw data are available upon request.

## Supplementary Materials

The following supporting information can be downloaded at: https://www.mdpi.com/article/10.3390/nu14245355/s1, Table S1. Dietary intake of studied patients at baseline and endpoint.
