# Supplementary material for: Precision Nutrition in NAFLD: Effects of a High-Fiber Intervention on the Serum Metabolome of NAFD Patients—A Pilot Study
Source: Nutrients. 2022 Dec 16;14(24):5355. doi: 10.3390/nu14245355 (PMC9787857; doi:10.3390/nu14245355)
Supplement: Supplementary file 1 [file nutrients-14-05355-s001.zip › nutrients-2077373-supplementary.pdf]

Supplementary Table S1. Dietary intake of studied patients at baseline and endpoint.

Parameter	Timepoint 1 median (range)	Timepoint 2 median (range)	p-value from t-test	p- value	
Energy from protein (%)	21.49 (12.6 - 33.4),	21.81 (10.7 - 28),	0.76,	0.84	Paired t-test
Energy from fat (%)	31.8 (17.7 - 52.7),	33.56 (15.2 - 51.9),	0.52,	0.84,	Paired t-test
Energy from carbohydrates (%)	43.5 (21 - 61.3),	42.09 (20.8 - 69),	0.80,	0.84,	Paired t-test
Water (g)	1324.2 (471 - 2658.2)	1228 (444.9 - 3453.6),	0.10,	0.44,	Wilcoxon signed rank test with continuity correction
Total protein (g)	73.9 (47.4 - 133.7),	70.57 (47.7 - 01.7),	0.36,	0.84,	Paired t test
Fat (g)	50.9 (16.5 - 134),	51 (23.1 - 88.2),	0.40,	0.84,	Wilcoxon signed rank test
Carbohydrate (g)	174.4 (60.4 - 364.2),	160.4 (54.5 - 75.8),	0.59,	0.84,	Wilcoxon signed rank test with continuity correction
Starch (g)	73.1 (21 - 159.2),	79.36 (22.6 - 180.8),	0.68,	0.84,	Paired t-test
Poliunsaturated Fatty acids (PUFA) (g)	0.2 (0 - 5.2),	0.3 (0 - 2.8),	0.63,	0.84,	Wilcoxon signed rank
Cholesterol (mg)	288.86 (113.5 - 619),	265.8 (127.6 - 652.4),	0.75,	0.84,	Wilcoxon signed rank test
Sodium (mg)	2007.9 (1063.2 - 4004.1),	2157.9 (970.1 - 4832.3),	0.93,	0.93,	Wilcoxon signed rank test with continuity correction
Pottasium (mg)	3245.8 (1883.8 - 5195.4),	2846.42 (1356.6 - 4833),	0.06,	0.11,	Wilcoxon signed rank test with
Calcium (mg)	619.2 (131.8 - 1893),	585.39 (148.5 - 1153.8),	0.49,	0.84,	Paired t-test

Phosphorus (mg)	1207.64 (662.3 - 2223.1),	1115.18 (703.4 - 1612.7),	0.18,	0.61	,Paired t-test
Magnesium (mg)	283.64 (149.7 - 506.7),	250.14 (116.2 - 461.1),	0.07,	0.39,	Paired t-test
